# Comparative label-free proteomics of the neonatal meningitis-causing *Escherichia coli* K1 IHE3034 and RS218 morphotypes

**DOI:** 10.1128/mra.00960-23

**Published:** 2024-01-30

**Authors:** Nikola Zlatkov, Wilma Gunnari, Ulrike Resch

**Affiliations:** 1 Department of Molecular Biology, Umeå Centre for Microbial Research (UCMR), Umeå University, Umeå, Sweden; 2 Department of Vascular Biology and Thrombosis Research, Center of Physiology and Pharmacology, Medical University of Vienna, Vienna, Austria; University of Guelph, Guelph, Ontario, Canada

**Keywords:** proteomics, newborn meningitis, NMEC, ExPEC

## Abstract

The proteome of two newborn meningitis *Escherichia coli* K1 (NMEC) morphotypes was examined via a label-free proteomics approach. Besides shared NMEC virulence factors, the two strains have different evolutionary strategies—strain IHE3034 tends to perform anaerobic respiration continuously, while strain RS218 maintains its filamentous morphotype due to active SOS response.

## ANNOUNCEMENT

**Fig 1:**
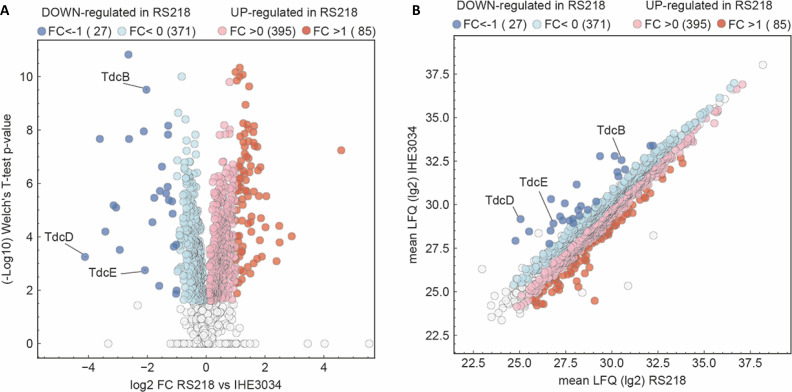
Detected protein species in RS218 and IHE3034. (**A**) Volcano plot displaying the upregulated (in blue) and downregulated (in red) proteins in RS218 as compared to ones of IHE3034, statistically non-significant proteins are shown in gray. (**B**) Abundance of the expressed proteins detected in RS218 and IHE3034.

The neonatal meningitis-causing *Escherichia coli* O18:K1:H7 (NMEC) is the leading Gram-negative agent of newborn meningitis and onset sepsis, and one of the deadliest (1, 2). The strains of this serotype are part of the very versatile phylogenetic B2 group *E. coli* pathovars, collectively referred to as extraintestinal pathogenic *E. coli* (ExPEC) (3). In addition to the common for ExPEC virulence factors including virulence-associated fimbriae, siderophores and cytotoxins, a typical neuroinvasive *E. coli* assembles S-fimbriae, displays invasion-facilitated transcytosis, and belongs to the K1 capsular serotype (2). *E. coli* IHE3034, a Finnish neonatal meningitis isolate, and the American isolate RS218 are a case in point (4, 5). Even though these strains share the same virulence and serotype properties, we found that they belong to different morphotypes—while RS218 cells are filamentous, IHE3034 cells are of normal cell size with a tendency to steadily perform anaerobic metabolism even in oxygenic environments (6, 7). To uncover the molecular basis of these complex phenotypes, RS218 and IHE3034 bacteria were grown on solid lysogeny medium and subjected to comparative, label-free quantitative (LFQ) proteomics (6). The following sample preparation and analysis methods are an expanded version previously described in our related work (6). Prior to the proteomics analysis, the total soluble proteome of five replicates from 10^9^ cells of each strain was isolated after three cycles of freeze thawing in lysis buffer, and 200 μg was subjected to chloroform/methanol precipitation as described in reference 8. Proteins were solubilized and denatured in 2M Urea, reduced (10 mM DTT) and alkylated (50 mM IAA), and digested with trypsin according to the manufacturer’s protocol (Promega). Acidified tryptic digests were desalted and concentrated with C18 SPE columns (Thermo Scientific) and subjected to nano LC-MS/MS analysis using an Ultimate 3000 nano UHPLC system coupled to a Q Exactive HF mass spectrometer (Thermo Fisher Scientific) with an ESI nanospray source. The LC conditions were a flow rate of 250 nL/min and a stepwise linear gradient made with 0.1% formic acid aqueous and acetonitrile organic phase. The gradient was 2% to 8% for 3 min, 8% to 20% for 60 min, 20% to 40% for 33 min, and 40% to 90% for 4 min. The full MS scan was conducted between 300 and 1,650 m/z at the resolution of 60,000 at 200 m/z. The raw MS files were processed using Maxquant (1.6.2.6) (9) against the *Escherichia coli* K1 Uniprot reference proteome, default search parameters (trypsin specificity with one missed cleavage, fixed C-carbamidomethylation, variable M-oxidation, 10 ppm MS, and 0.5 Da MS/MS tolerance) and LFQ enabled. Initially, the protein species detected in every sample were subjected to cluster analysis to identify mutually exclusive unknown groups. The protein expression matrix was transformed by -log10; its values were clustered by hierarchical clustering in Genesis (1.8.1) (10) and visualized using the “heatmap.2” function from the “gplots” R-package (11). For statistical analysis of differentially expressed proteins in RS218 versus IHE3034, unpaired two-tailed Welch’s *t*-test with Benjamini-Hochberg FDR correction (*P* < 0.05) was applied on log2-transformed LFQ-intensity values in Perseus (v 1.6.15.0) (12). Volcano plots showing log2-transformed fold changes (FC) of RS218 versus IHE3034 on *x*-axis and -log10 *P*-value on *y*-axis were prepared in Instant Clue (v 0121) with significant proteins (FDR corrected *P* < 0.05) color coded for FC <0 (blue) or FC >0 (red), see Fig. 1A (13). Scatter plots of log2-transformed mean LFQ intensities were prepared to illustrate protein abundances of significantly regulated proteins (Fig. 1B). The downstream analysis of the proteomics data includes Gene Ontology (GO) (http://www.geneontology.org/) and KEGG (http://www.genome.jp/kegg/) annotation and analysis, as well as String (http://string-db.org/) functional protein interaction analysis (14, 15). Gene Ontology annotation proteome was derived from the UniProt-GOA database (http://www.ebi.ac.uk/GOA/). First, the identified protein IDs were converted to their UniProt IDs and then mapped to GO and KEGG IDs. The GO and KEGG pathways with an enrichment *P*-value (obtained from Fischer exact test with Benjamini-Hochberg correction) were considered significant when *P* < 0.05. To define protein-protein interactions, String database (http://string-db.org/) was used for the differentially expressed proteins with combined score 0.4 as a threshold value. Default parameters were used for all the programs in this study except where otherwise noted. In this study and as a primary outcome, we identified 1,363 protein species, as 309 were differentially produced—162 upregulated and 148 downregulated in RS218, and after FDR-adjustment, 112 proteins displayed differential pattern—27 downregulated and 85 upregulated (Fig. 1).

## Data Availability

The mass spectrometry proteomics data have been deposited to the ProteomeXchange Consortium via the PRIDE (16) partner repository with the data set identifier PXD036163.
